# Equivalent Indels – Ambiguous Functional Classes and Redundancy in Databases

**DOI:** 10.1371/journal.pone.0062803

**Published:** 2013-05-02

**Authors:** Jens Assmus, Jürgen Kleffe, Armin O. Schmitt, Gudrun A. Brockmann

**Affiliations:** 1 Breeding Biology and Molecular Genetics, Humboldt-Universität zu Berlin, Berlin, Germany; 2 Institut für Molekularbiologie und Bioinformatik, Charité Berlin, Berlin, Germany; Université de Nantes, France

## Abstract

There is considerable interest in studying sequenced variations. However, while the positions of substitutions are uniquely identifiable by sequence alignment, the location of insertions and deletions still poses problems. Each insertion and deletion causes a change of sequence. Yet, due to low complexity or repetitive sequence structures, the same indel can sometimes be annotated in different ways. Two indels which differ in allele sequence and position can be one and the same, i.e. the alternative sequence of the whole chromosome is identical in both cases and, therefore, the two deletions are biologically equivalent. In such a case, it is impossible to identify the exact position of an indel merely based on sequence alignment. Thus, variation entries in a mutation database are not necessarily uniquely defined. We prove the existence of a contiguous region around an indel in which all deletions of the same length are biologically identical. Databases often show only one of several possible locations for a given variation. Furthermore, different data base entries can represent equivalent variation events. We identified 1,045,590 such problematic entries of insertions and deletions out of 5,860,408 indel entries in the current human database of Ensembl. Equivalent indels are found in sequence regions of different functions like exons, introns or 5' and 3' UTRs. One and the same variation can be assigned to several different functional classifications of which only one is correct. We implemented an algorithm that determines for each indel database entry its complete set of equivalent indels which is uniquely characterized by the indel itself and a given interval of the reference sequence.

## Introduction

The increasing use of next-generation sequencing (NGS) during the last years has caused the discovery and registration of millions of human sequence variations in genome databases such as the database of the National Center for Biotechnology Information (NCBI) [1] or the Ensembl database [2]. In the beginning of the NGS era (2000–2005), neither the correctness nor the novelty of the variations submitted to a database were carefully checked. Thus, many variations were newly registered although they were already known. Some published variations were even found to be in conflict with the reference sequence.

Now, improved submission management systems make a quality-control and formally check if a genetic variation that is submitted to a database is identical with an already existing database entry or if it is novel [3], [4]. In the case of substitutions (i.e. one allele is replaced by another or a stretch of alleles is replaced by another stretch of the same length) it is straightforward to decide if a submitted variation is novel or if it coincides with an already existing one. Any substitution differing in alternative allele or in position from a documented variation is novel.

The situation is somewhat more complex for indels. Whereas identity of reference and alternative alleles as well as start and end position with those of an existing entry is sufficient to reject a submission as novel, formal divergence in these characteristics does not guarantee its novelty. The reason is that in many cases an insertion to the reference sequence or a deletion from it can be realized in multiple ways such that one and the same alternative sequence result.

As a very simple example we consider the short reference sequence CAAGT. The deletion of the first or the second A would result in the same alternative sequence CAGT. Such a deletion would be denoted as [A/−]. In the first case, the position of the indel would be annotated as 2–3 (C[A/−]AGT), in the latter case as 3–4 (CA[A/−]GT). Although in the original mutation event either the first or the second A was lost, the two cases cannot be distinguished any more. Biologically, the two cases are completely equivalent because they result in the same alternative sequence. There is currently no convention that avoids publication of both indel annotations if submitted from different research groups. Therefore, sometimes one of several possibilities, sometimes more than one (but not all), sometimes all possible annotations are represented in a database.

In this work we present an algorithm that is able to identify the complete list of alternative annotations for any given indel, which we call the complete set of equivalent indels. All these indels generate the same alternative sequence and, hence, have the same biological effect. We apply the algorithm to all indels that are presently available in the Ensembl database and show that their number can be considerably consolidated.

Within the 1000 genomes project, researchers found variations which formally differ, but which lead to the same modified sequence, i.e. they are biologically equivalent. Hence, annotating the two indels as different indels would cause redundancy in genome variation databases. To avoid resulting redundancy, two variations were considered equivalent if their alleles have the same length and if both occur in a distance of less than 25 base pairs [5]. This method reduced the redundancy significantly but not completely. It also introduced errors by always considering indels redundant which are located close to each other.

A correct but slow algorithm to detect equivalence was developed in 2010 [6]. It repeatedly compared a given indel with the directly neighbouring sequence section of the same length constituting an alternative indel. It checked whether the allele of the alternative indel was a cyclic permutation of the allele of the given indel. Based on some more mathematics, the algorithm presented in this paper determines the set of all equivalent indels by direct calculation and without sequence permutation. This improvement is important for identifying long intervals of equivalent indels.

The exact position of a variation is also important in phylogenetics to calculate homology. Ambiguous indels might lead to different taxonomies [7].

Ambiguous variation is a similar problem to ambiguous alignment [8]. Nevertheless, there are some differences: If several reads can be aligned in multiple ways, this is called ambiguous alignment, but the resulting alignments are not necessarily equivalent. The resulting sequences can differ. Thus, the ambiguity of ambiguous alignments can be resolved (at least theoretically) by extending the length of the reads. If the reads were long enough, there would be no more ambiguity. For example, a read matches perfectly at two different genomic loci. Then, there are two possible alignments, one for each genomic locus. Yet, an extension of the read would clarify which alignment is the correct one, i.e. which genomic locus was sequenced. This disambiguation is not possible for ambiguous indels. Even if a read had the length of a whole chromosome, a comparison of the read with the reference sequence would lead to ambiguous indels. Therefore, in contrast to an ambiguous alignment, it does not make sense to speak of the correct or incorrect annotation of an indel. Ambiguous indels are equivalent to each other.

Let us begin with introducing some definitions about genetic variations and their properties which are used in the course of this paper.

1. A *sequence variation*, or alternatively a *genetic variation*, is a substitution, deletion, or insertion of one uninterrupted piece of sequence of a given length at a given position of the reference sequence. It results in a changed sequence which is called *alternative sequence*.
2. Two genetic variations are called *equivalent* if and only if both generate the same alternative sequence and hence the same genotype. Necessarily, equivalent variations have equal length.
3. A genetic variation is called *ambiguous* if and only if there exists an equivalent variation at an alternative position of the reference sequence (Table 1). Hence, an ambiguous variation is just one out of a number of equivalent variations.
4. The *region of ambiguity* (RoA) for a specific variation is the region where equivalent variations can occur.
5. The *degree of ambiguity* of a specific variation is the number of possible equivalent variations.

**Table 1:** Different variations lead to the same alternative sequence and, therefore, the variations are equivalent.

	aligned sequences	unaligned sequences	
reference sequence	TTGCAAAAAAAAAA***ATGCCTA***	TTGCAAAAAAAAAAATGCCTA	
*rs34061715*	TTGCAAAAAAAAAA ***TGCCTA***	TTGCAAAAAAAAAATGCCTA	(start lost)
equivalent deletion	TTGCAAAAAAAAA ***ATGCCTA***	TTGCAAAAAAAAAATGCCTA	(no effect)
equivalent deletion	TTGC AAAAAAAAA***ATGCCTA***	TTGCAAAAAAAAAATGCCTA	(no effect)

The first variation is a deletion at position 15, the second variation is a deletion at position 14, and the third variation is a deletion at position 5.

This deletion is annotated in Ensembl as lying in the start codon of transcript HRNR-001 and therefore leads to the loss of the start codon. The equivalent indel has no effect on the protein. (Sequences are all shown as reverse complementary, because the transcript is located on the reverse strand.) Regular characters denote the upstream region and bold, italic characters the coding sequence.

A set of indels which are all equivalent to each other forms an equivalence class of biologically identical variations. The entries of this set represent only different annotations of the same indel.

## Methods & Algorithms

### 1 Scanning the Whole Human Database

We used the *Ensembl variation database* (v70) for human data for our analysis. This database is based on *dbSNP* [9] and includes data from 21 further sources (e.g. COSMIC [10], OMIM [11], UniProt [12]). It is accessible via SQL. For each variation of the database the following entries were used in our analysis: the variation identifier rsID, chromosome number, start, end, reference allele, alternative allele, and functional class. Variations which failed the Ensembl Quality Control check [3] were excluded from further analysis. Each remaining human variation entry was tested for ambiguity using an algorithm that is described below. The package BioPerl [13] and the Ensembl API [4] were used to download the human genome (sequences, transcript annotation and variations) from the Ensembl database. A short overview of the analysis is shown in the PRISMA Flowchart S1.

First, all possible alternative positions of the variations were determined. Then, NovelSNPer [14] was used to get the formal functional classes of all equivalent variations. We checked whether the functional classes differed between equivalent indels. We also checked equivalences between all annotated variation entries.

### 2 Determining the Region of Ambiguity for Deletions

Our method of deriving the interval of equivalent deletions begins with considering neighbouring deletions, i.e. subsequences of the same length which start at neighbouring positions of the reference sequence. This simplest case is covered by the following Theorem 1 that needs no proof.

**Theorem 1.** *Let 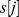 denote the j-th nucleotide of the reference sequence and 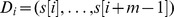 be a deletion subsequence of length 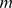.*

**Figure 1:**
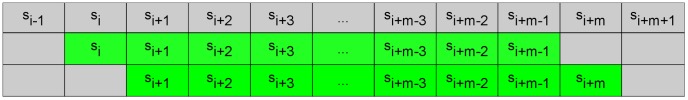
Illustration of Theorem 1. The first line is the reference sequence. The second and third lines contain two deletions. 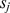 are the nucleotides. If 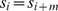, then the two deletions are equivalent.

1. *The downstream neighbouring subsequence 
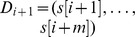
 represents an equivalent deletion if and only if 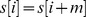 i.e. the first nucleotide of the first deletion coincides with the last nucleotide of the second deletion. (see* *Fig. 1* *).*
2. *The upstream neighbouring subsequence 
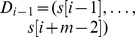
 represents an equivalent deletion if and only if 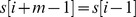.*

This theorem permits repeated testing for equivalence of downstream and upstream neighbouring deletion sequences until the test rejects equivalence. The result is an interval (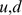) of the reference sequence that harbours equivalent deletions. The necessary computations are described by the pseudocode in Table 2. Table 3 illustrates how the interval (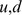) of equivalent downstream deletions for the deletion event TATT[ACGG/−]ACGGACTTG is derived.

**Table 2:** Code 1: Pseudocode for the identification of the region of ambiguity for deletions.

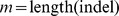
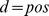 ; 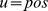 ;
**while** 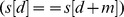 {
print( 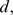 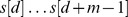 );
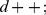
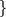
**while** 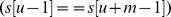 {
print( 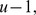 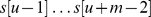 );
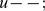
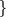
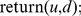

The variable 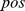 is the start position of the deletion, 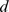 is the most downstream start position of equivalent deletions, 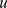 is the most upstream start position, and 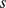 is the reference sequence. All possible variations are printed in a file for reference purposes and the most upstream and downstream start positions 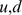 are returned.

**Table 3:** An example of the algorithm code 1: A deletion sequence is shifted downstream to detect equivalent deletions.

reference sequence	TATTACGGACGGACTTG
original alignment	***ACGG*** :
1st alignment	***CGGA*** :
2nd alignment	***GGAC*** :
3rd alignment	***GACG*** :
4th alignment	***ACGG*** :
5th alignment	***CGGA*** :
6th alignment	***GGAC*** :
mismatch	***GACG***

The deletion sequence is printed in bold italic. The nucleotide following the deletion sequence is compared with the first nucleotide of the deletion. If both are equal, the variation is shifted 1 bp downstream, otherwise the algorithm terminates. In our example the algorithm terminates after the 6th alignment.

It remains to be shown that the set of subsequences 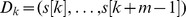 for 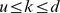 constitutes the complete set of all deletions that are equivalent to 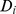. This is proven in two steps of which the first considers a pair of overlapping deletions.

**Theorem 2.** *Let 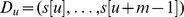 and 
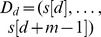
 for 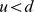 be two overlapping equivalent deletions.*

*Then* 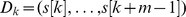 *for* 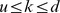 *are all equivalent deletions.*

*Proof:* Let us decompose the overlapping sequences 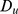 and 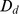 into the uniquely defined subsequences 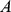, 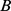, and 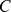 such that 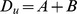 and 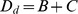 where 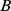 denotes the overlapping part of 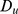 and 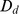. Then equivalence of 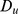 and 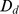 implies 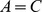, and from Theorem 1 follows that all downstream neighbours of deletion 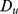 until 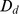 are equivalent deletions. The case of adjacent equivalent deletions 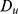 and 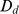 is covered by 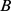 being the empty sequence and it follows 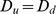.

The second step extends Theorem 2 to distantly located equivalent deletions that do not overlap.

**Theorem 3.** *Let 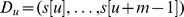 and 
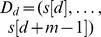
 for 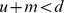 be two distantly located equivalent deletions. Then 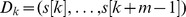 for 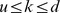 are all equivalent to the deletions 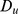 and 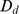.*

*Proof:* Let 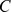 denote the sequence located between 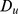 and 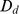. Then equivalence of 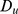 and 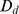 implies 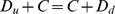, i.e. the sequence section 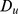 extended by sequence section 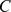 must be the same as sequence section 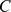 extended by 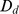.

**Figure 2:**
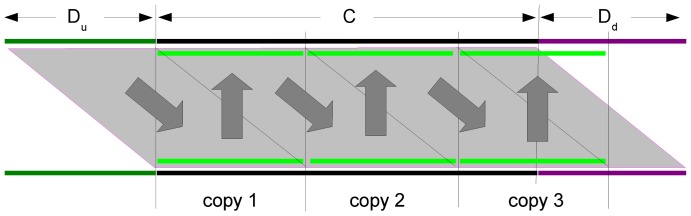
Illustration of the proof of Theorem 3. Shown are two aligned copies of the section 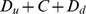 of the reference sequence where 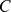 (black) represents the sequence between two equivalent deletions 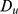 (green) and 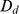 (magenta). The filled polygon illustrates the sequence identity 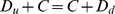 that holds if and only if 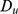 and 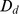 are equivalent. Following the grey arrows up and down and from left to right, it can be seen that the sequence section 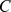 consists of repeats of the deletion sequence 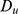 until the last copy overlaps with the deletion sequence 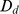.

Figure 2 shows two copies of the section 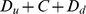 of the reference sequence where the filled polygon marks the two identical subsequences 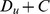 and 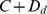. As indicated by the leftmost grey arrow we conclude that the sequence section 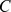 starts with a copy of 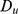 called copy 1. Repeatedly using this argument proves that the sequence section 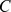 is formed by repeats of 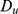 until a last repeat (copy 3 in Fig. 2) overlaps with the sequence section 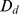. All these copies are equivalent to the deletion 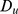 and it follows from the transitivity property of the equivalence relation that this last copy of 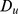 is equivalent to 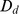. Hence, the entire interval beginning at the start of 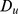 and ending with the start of 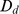 is covered by overlapping equivalent deletion sequences. Finally, Theorem 2 says that each nucleotide in this interval is the start position of an equivalent deletion.

Summarizing, we have proved that for any given pair of equivalent deletions all positions between the two provide equivalent deletions, too. Therefore, complete sets of equivalent deletions are always defined by deletion intervals bounded by the most upstream equivalent deletion 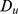 and the most downstream equivalent deletion 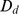, which are easily found by performing the calculations described in Table 2. Moreover, deletion intervals are always formed by the union of two repeat regions obtained by all downstream repeats of 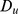 and all upstream repeats of 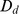. Neither 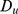 nor 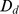 must have a complete repeat in the reference sequence but they must fit together in well defined way. For example, the two deletions CCC[ATG/−]ATCCC and CCCAT[GAT/−]CCC are equivalent and the interval of equivalent deletions CCC(ATGAT)CCC contains ATG and GAT only ones. However, the sequence section ATGAT is periodic with periodicity 3. The algorithm described in Theorem 1 proves periodicity for each interval of equivalent deletions with periodicity equal to the length of deletion.

### 3 Determining the Region of Ambiguity for Insertions

We developed also a simple algorithm for identifying all equivalent insertions. Let us call the reference sequence 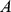 and for a given insertion the alternative sequence 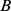. First, perform the given insertion on the reference sequence. Identify the resulting sequence as new reference sequence and identify the former reference sequence as new alternative sequence. In other words, switch the role of reference sequence and alternative sequence, i.e. the sequence 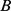 is the new reference sequence and the sequence 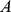 is the new alternative sequence. Thus, the insertion in the former reference sequence 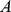 becomes a deletion in the new reference sequence 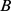. Afterwards, identify all equivalent deletions. If two deletions are identified as equivalent by the algorithm, the two deletions have the same alternative sequence. The reference sequence is also the same by definition. Therefore, the role of sequences can be switched again, i.e. the current reference sequence 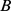 is again the alternative sequence and the current alternative sequence 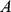 is again the reference sequence. Thus, the two deletions become insertions, which are still equivalent.

A disadvantage of this method is that the reference sequence has to be changed for performing the calculations of Table 2. Let us therefore propose a modified code that directly compares the letters of the insert sequence with those of the unchanged reference. This algorithm handles insertions and deletions in the same way by using the periodicity of the region of ambiguity.

Instead of comparing two nucleotides of the reference sequence, it compares one nucleotide of the reference with the corresponding nucleotide of the allele.

A pseudocode of this algorithm is shown in Table 4. An example of an implementation in Perl can be downloaded at http://www2.hu-berlin.de/wikizbnutztier/software/Equivalence/.

**Table 4:** Code 2: Pseudocode for the identification of the region of ambiguity for all indels.

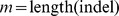
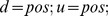
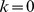 ;
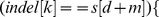 **while**
print( 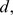 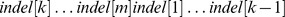 );
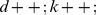
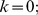 **if** 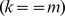
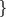
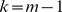 ;
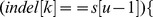 **while**
print( 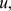 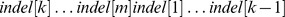 );
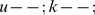
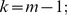 **if** 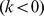
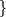
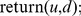

The variable 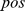 is the start position of the deletion, 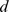 is the most downstream start position of equivalent indels and 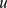 is the most upstream start position. Compared to the code of Table 2 the index 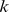 now cyclicly provides the nucleotide indel[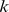] directly from the indel allele.

### 4 Model

Our theoretical results not only allow to find all sequence variations witch are equivalent to a given variation but also draw a clear picture how complete sets of equivalent variations look like.

1. All ambiguous variations are indels.
2. If two deletions are equivalent, then all deletions of the same length and located between the two are also equivalent to each other.

If two insertions are equivalent, then there exists an equivalent insertion at each position between the two.

1. All equivalent indel sequences are cyclic permutations of each other.
2. If two deletions have the same length and the same region of ambiguity, these two deletions are equivalent.
3. Two indels with the alternative alleles 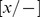 respectively 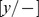 are equivalent if they lie in the region of ambiguity of each other and their alleles fulfil the equation 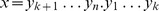, where 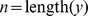 and 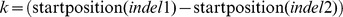 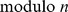.
4. The region of ambiguity is an interval of the form 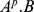, where 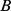 is a prefix of 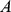 and 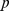 is the length of the ambiguous indel.
5. The degree of ambiguity for deletions is equal to the length of the region of ambiguity minus the length of the deletion.

The degree of ambiguity for insertions is equal to the length of the region of ambiguity.

1. Ambiguous variations can occur in non-repetitive sequences although most of them occur in repetitive sequences.

## Results

### 1 Analysis of Ensembl Database

**Figure 3:**
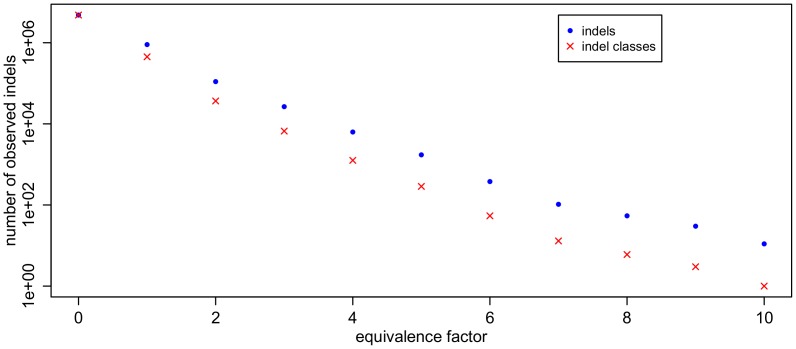
Number of indels in the human database (Ensembl v70) versus the equivalence factor. Blue dots represent numbers of indels and red crosses represent numbers of ambiguity intervals, i.e. classes of equivalent indels. The equivalence factor indicates the number of equivalent variations for each variation. This means, an indel of equivalence factor 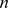 has 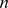 further equivalent entries in the database.

The Ensembl variation database entries are split into those called *Synonyms* for which equivalent variations are known and others called “without synonyms”. We found several human indels which are labelled “without synonyms”, but nevertheless have equivalent variations in the database (see Table S1). This is a formal database error. The majority of 4,814,818 indels are unique. The rest of 1,045,590 indels are ambiguous and can be consolidated to 495,149 equivalence classes as shown in Fig. 3 and Table S2. On the other hand, there are generally very few representatives of ambiguous indels published in the database. We conjectured that short indels are more frequently ambiguous and have a higher degree of ambiguity. But our analysis did not reveal significant correlation between the length of an indel and its degree of ambiguity, which indicates the number of possible equivalent variations. Most equivalent variations are located close to each other, but distances of up to 157 nucleotides were observed (*rs6145932* and *rs11272715*).

**Figure 4:**
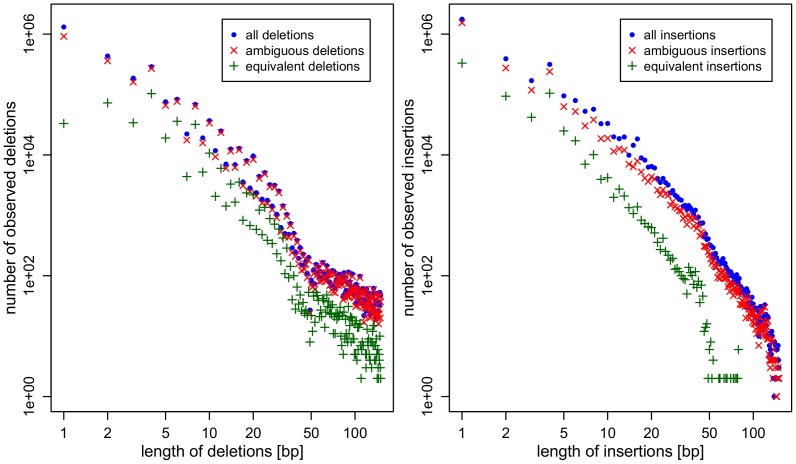
Number of observed insertions (a) and deletions (b) versus length. There is strong correlation between length (x-axis) and frequency (y-axis) of the indels.

The number of long ambiguous indels is lower than the number of short ambiguous indels. This might be due to the fact that long indels are generally less frequent than short indels (Fig. 4).

The supplementary data (File S1) lists all ambiguous indels with a degree of ambiguity of at least 10 for which at least 5 database entries exist currently.

### 2 List of Examples of Ambiguous Indels

This section lists some examples to give an overview of the diversity of ambiguous indels.

**Figure 5:**
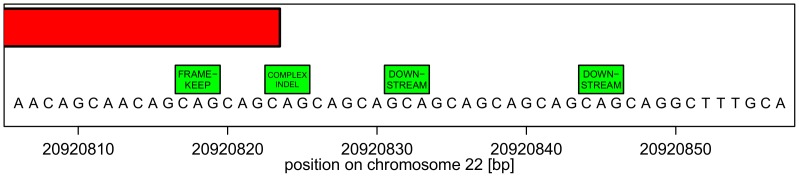
An ambiguous deletion with formally different functional classification. *rs71769813* is a deletion of CAG located in the MED15 gene (mediator complex subunit 15) on human chromosome 22 at position 20,920,823 (forward strand). The red box is the exon and the green boxes are deletions. Equivalent deletions are located in the coding region, at the transcription end, or downstream of the transcript MED15-203.

**Figure 6:**
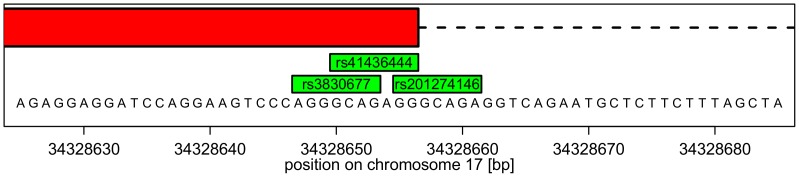
The position of a deletion in a repetitive sequence depends on the alignment of the alternative sequence. There is a deletion of AGGGCAG located in RP11-104J23.1-001 on human chromosome 17. This deletion occurs three times in the dbSNP database. They are represented by green boxes; each time with a different identifier, a different position, and different functional classes. The red box represents an exon of the transcript.

- **Simple example with indel of length 1.** A simple example is the human mutation *rs11450129* [−/C] that reports the insertion of a single nucleotide C between positions 19,810,810 and 19,810,811 of the human chromosome 1. The surrounding reference sequence GGTGG[−/C]CCCCCCAAGG tells us that the nucleotide C could as well be inserted 1, 2, 3, 4 or 5 positions downstream without affecting the alternative sequence. In fact, the database entries *rs66548569*, *rs72506980*, and *rs58967920* provide such equivalent variations. These indels are all equivalent. The unique alternative sequence GGTGGCCCCCCCAAGG has one more C in its run.
- **Multiple functional classes.** In addition, there are much more complex cases, too. One such case is the human mutation database entry *rs71769813* [CAG/−]. It represents a CAG deletion located in the MED15 gene (mediator complex subunit 15). The mediator complex is necessary for the expression of protein-coding genes [15], [16]. Fig. 5 shows the reference sequence around the deletion that is part of a 12-fold CAG repeat region. It is easily seen that the deletion of any nucleotide triplet GCA, CAG or AGC within this region generates the same alternative sequence. The annotated variation is ambiguous with 34 equivalent deletions.
- Moreover, the repeat region contains the transcription end so that some equivalent deletions are in the coding region or downstream of the transcript. Here, an ambiguity in the position of the indel entails ambiguity in the functional classification. The change of genotype is always the same: a CAG repeat that is one repeat unit shorter irrespective of which triplet is removed. This unique change is a copy number variation or repeat disorder with or without effect.
- **Ambiguous indel affecting the start codon.** Also start codons can be affected by the ambiguity of variations: *rs80268284* at position 152,195,729 on human chromosome 1 is located at the start of the HRNR-001 transcript (reverse strand). This deletion is annotated as FRAMESHIFT_CODING and START_LOST. A closer look at the neighbouring sequence indicates that it probably does not have an influence on the protein structure as shown in Table 1.
- **Region of ambiguity in non-repetitive sequence.** An example of a region of ambiguity in non-repetitive sequence is *rs5904359* at position X:152,610,226–152,610,234 with the alleles [TGACCTCTG/−] (see Table 5). Our algorithm identified a region of ambiguity of length 4. Thus, there are three equivalent variations. These are in fact published as *rs35024993*, *rs5904360*, and *rs3047915*.
- **Equivalent variations involved in a triplet-repeat disease.** Triplet-repeat diseases often consist of variations, which are annotated multiple times. For example, *Huntington’s disease* is a triplet-repeat disease which breaks out if the triplet-repeat-region CAG in the first exon of the gene HTT is too long [17]. The normal length used as consensus sequence comprises 20 repeats. Two published insertions, which are equivalent, reduce the CAG-repeat-region: *rs72457839* and *rs71180116*.
- **Parkinsonism and ataxia.** A similar case are the human mutations *rs201732168*, *COSM247745*, *rs113202486*, and *rs71010672* located in the TATA-box binding protein on chromosome 6. Here again, a 19 fold CAG repeat covers the mutation site. Each of the four variations extends the repeat. The imposed triplet disorder is known to cause parkinsonism and ataxia [18], [19].
- **Indels affecting splice sites.** Another example is a 7 base pair deletion in the region 17:34,328,647–34,328,663. The reference sequence is AGGGCAGAGGGCAGAGG and the alternative sequence is AGGGCAGAGG. There are three annotations of this deletion (*rs3830677*, *rs41436444*, and *rs201274146*) with different functional classes (Fig. 6).
- **Ambiguous insertion affecting a start codon.** To complete the list of examples we also shortly discuss the insertion event *rs55710688* [−/CCCA] that inserts the sequence CCCA between the leading A and the ending TG of the start codon of the transcript WNT16-002 (wingless-type MMTV integration site family, member 16). It formally received the functional classifications FRAMESHIFT and START_LOST in Ensembl. But a closer look at the reference sequence context reveals that this insertion maintains the start codon as seen in Table 6. Inserting CCCA four positions upstream results in the same alternative sequence demonstrating the ambiguity of the insertion.

**Table 5:** An example of equivalent deletions in a non-repetitive sequence on human chromosome 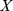.

rsID	start	End	deletion	alt. sequence
reference sequence	152610223	152610238	ACGTGACCTCTGTGGG	ACGTGACCTCTGTGGG
*rs35024993*	152610225	152610233	AC***GTGACCTCT***GTGGG	ACGTGGG
*rs5904359*	152610226	152610234	ACG***TGACCTCTG***TGGG	ACGTGGG
rs5904360	152610227	152610235	ACGT***GACCTCTGT***GGG	ACGTGGG
rs3047915	152610228	152610236	ACGTG***ACCTCTGTG***GG	ACGTGGG

The deletions are bold and italic. All four variations are equivalent although they are located in a non-repetitive sequence. All four variations are annotated in dbSNP. The alternative sequence is in each case ACGTGGG.

**Table 6:** Different functional classes of insertion *rs55710688* on human chromosome 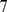 at position 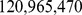.

reference sequence	AGGCACCCa tgcagctc
annotated insert	AGGCACCCa***ccca***tgcagctc
equivalent insert	AGGCA***CCCA***CCCatgcagctc
reference sequence	AGGCA CCCatgcagctc

The coding region is shown in lower case. The insertion *rs55710688* is bold italic. Lines 1 and 2 represent the alignment as annotated in dbSNP: The insertion lies inside the coding region and causes a start lost. Lines 3 and 4 represent an alternative alignment: The insertion lies outside the coding region. The insertion does not affect the start codon ATG.

## Discussion

A variation can have different functional classes for alternative transcripts if it is located in a gene with several transcripts. Such a variation has a single entry in the variation database of Ensembl, but the information about the functional class is available for each transcript separately. In this paper we did not study ambiguity arising from different transcripts of one gene, but ambiguity that is arising from ambiguous positions of indels. There may be several annotated functional classes for one indel, even though there is only one transcript. The question is, how to handle ambiguity in databases.

On the one hand, there are millions of variations reported in mutation databases. Reducing this number by deleting equivalent variations would be beneficial. This would also remove the bias in various analyses, e.g. when searching for regions with a high mutation rate. A repetitive region may have only one ambiguous mutation, but due to different alignments these equivalent mutations are reported several times at different positions and the region is incorrectly assumed to have a high mutation rate. Representing all equivalent variations by a single database entry would avoid this problem.

On the other hand, variation analysis tools would predict different functional classes and draw different conclusions depending on ambiguously positioned variations. For example, the deletion *rs3830677* is located in the exon of the transcript RP11-104J23.1-001, but the equivalent deletion *rs201274146* is in the intron of this transcript (Fig. 6). Therefore, it would be helpful if all equivalent indels were available in the database.

A synthesis of these two points of view would be the representation of a complete set of equivalent variations by its most upstream variation and the length of the region of ambiguity.

The knowledge about the sequence structure of the region of ambiguity will not only help in variation-calling but also in building the correct alignment: If the reference sequence has the sequence 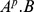, where the length of 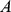 is the length of a gap and 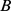 is a prefix of 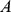 (see Table 7), the alignment tool can be restricted to one alignment and does not have to check all possible alignments, because they are equivalent. This would speed up the alignment process.

**Table 7:** Example of the prefix in a sequence.

reference sequence	AGGCATTCATTCATTCATT***CA***GGA
alternative sequence	AGG CATTCATTCATT***CA***GGA

In this example, the missing sequence is 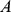 = CATT which is repeated 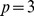 times and the prefix of the repeated sequence is 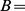
CA, which is shown in bold italic.

Scanning for ambiguous variations will also be beneficial for analysing repeat induced diseases (like trinucleotide disorders).

It might contribute to the understanding of this type of disease to analyse the cellular impact (e.g. functional classes) of these variations. The knowledge of all possible positions of an indel will help to determine the functional classes and understand the resulting change in the protein. It seems that such an insertion not only affects the length of the protein but also has a side effect: The polyglutamine disease is caused by insertions of CAA in the coding region and, therefore, enlarges the protein by glutamine. But an insertion of CAA is less toxic than an insertion of CAG [20], although both are translated to the amino acid glutamine. Thus, the insertion does not only affect the protein translation by adding glutamine. To understand this behaviour it is necessary to look at the multiple possible locations of the insertions and reflect the impact of a different splice site due to the insertion. Depending on the possible location of the indel, different functional classes might be annotated. It is most likely that only one annotated functional class is correct. Yet, our current knowledge about alternative splice sites and alternative translation starts is not sufficient to give a clear answer based on the genomic sequence. Thus, it would be an advantage to present all possible functional classes for ambiguous indels. This ensures that the correct functional class is among them. To reveal the correct functional class of an ambiguous variation, it is not sufficient to sequence longer genomic reads. Yet, for some ambiguous variations the functional class can be specified by sequencing the transcript. To reveal whether an ambiguous variation lies in the 5' UTR or in the start codon (e.g. Table 6), the protein must be sequenced.

The problem of ambiguity is not only a problem of short indels. Long indels are affected likewise.

The redundancy in variation databases is probably a result of different alignment algorithms: There are several possibilities to align a sequence that contains an ambiguous variation to the reference sequence. Some alignment tools place an insertion or deletion to the most upstream position while others place the insertion or deletion to the most downstream position or somewhere in between. All are correct alignments. Although the alignments differ, they all represent the same variation.

## Supporting Information

Table S1
**The table presents the number of indels of a specific equivalence factor.** The equivalence factor indicates the number of equivalent variations for each variation. This means, an indel of equivalence factor 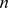 has 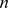 further equivalent entries in the database. An equivalence factor of 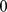 means that there exists no equivalent entry.(PDF)

Table S2 **The table presents the number of indels of a specific length.** In the column *#all ambiguous* the number of all ambiguous indels of this length are written. The column *#ambiguity classes* indicates, how many indels have the specific length if two equivalent variations are put together in one class.
(PDF)

File S1 **This file lists all ambiguous indels with a degree of ambiguity of at least 10 for which at least 5 database entries exist currently.**
(ZIP)

Checklist S1 **PRISMA Checklist.**
(DOC)

Flowchart S1 **PRISMA Flowchart.**
(TIF)
