# Correction: Equivalent Indels – Ambiguous Functional Classes and Redundancy in Databases

**DOI:** 10.1371/annotation/7e304601-fc5c-40fe-857c-d6ea894d1647

**Published:** 2013-12-19

**Authors:** Jens Assmus, Jürgen Kleffe, Armin O. Schmitt, Gudrun A. Brockmann

Multiple formatting errors were introduced into Tables 3, 6 and 7 in the preparation of this article for publication. Please view the correct tables here: 

**Figure:**
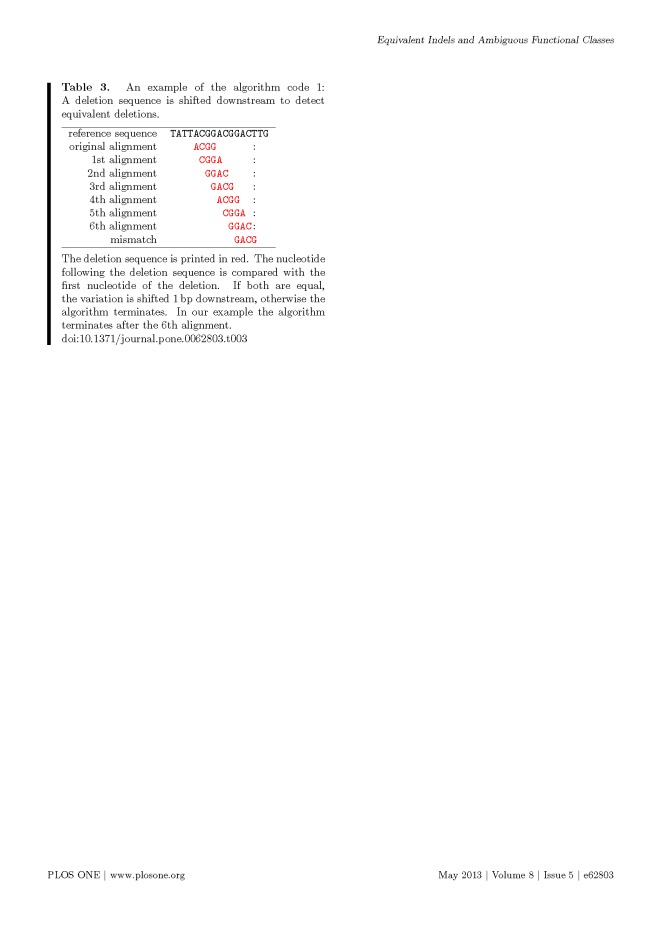


f 

**Figure:**
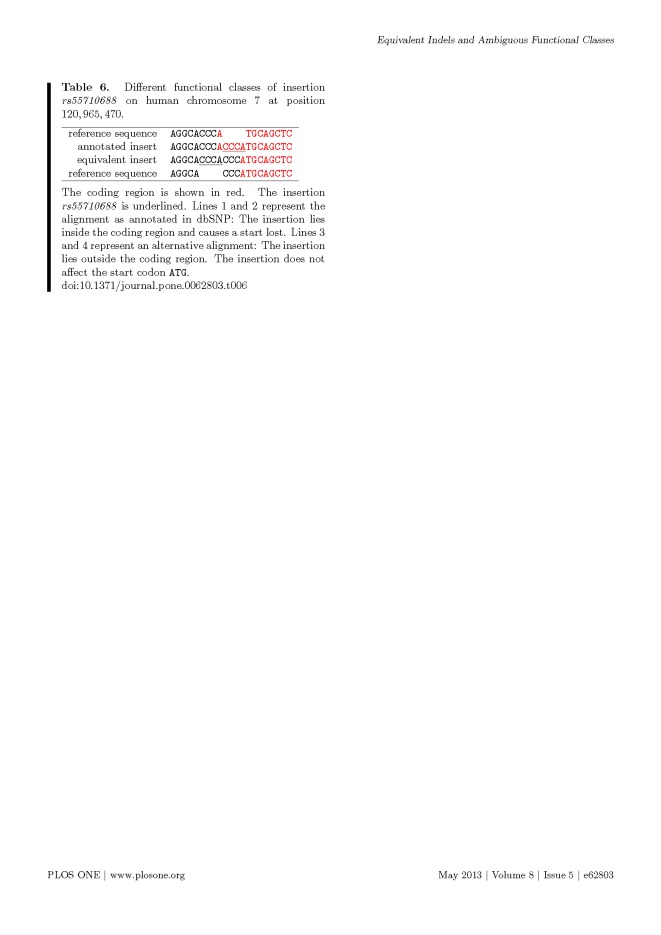


f 

**Figure:**
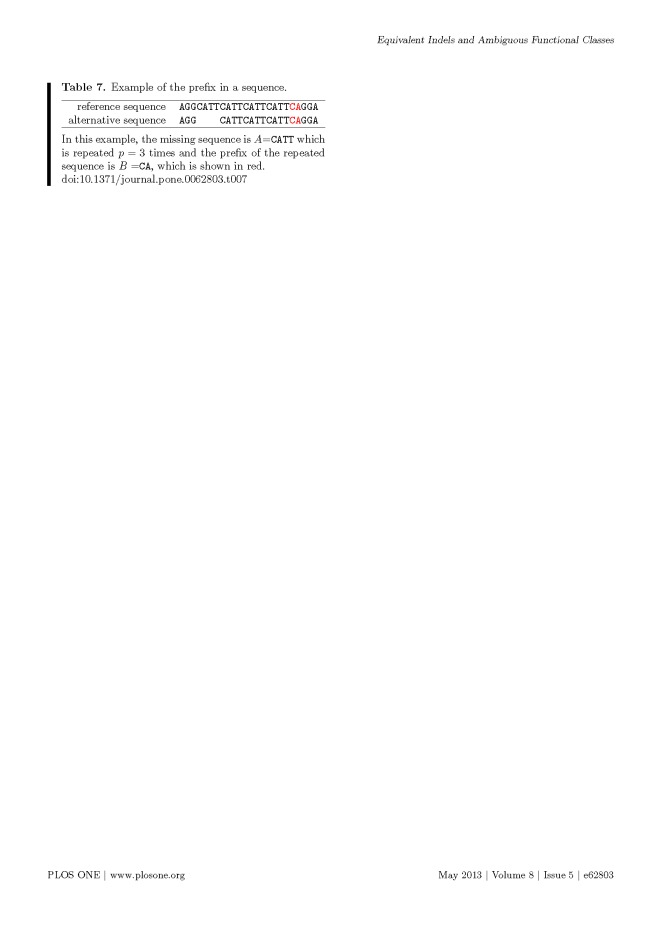


f

