# Supplementary material for: Equivalent Indels – Ambiguous Functional Classes and Redundancy in Databases
Source: PLoS One. 2013 May 2;8(5):e62803. doi: 10.1371/journal.pone.0062803 (PMC3642179; doi:10.1371/journal.pone.0062803)
Supplement: Table S1 — The table presents the number of indels of a specific equivalence factor. The equivalence factor indicates the number of equivalent variations for each variation. This means, an indel of equivalence factor has further equivalent entries in the database. An equivalence factor of means that there exists no equivalent entry. (PDF) [file pone.0062803.s001.pdf]

Table S1. The table presents the number of indels of a specific equivalence factor. The equivalence factor indicates the number of equivalent variations for each variation. This means, an indel of equivalence factor n has n further equivalent entries in the database. An equivalence factor of 0 means that there exists no equivalent entry.

	**Deletions**		**Insertions**	
equivalence factor	#all deletions	#ambiguity classes	#all insertions	#ambiguity classes
0	2 289 454	2 289 454	2 525 364	2 525 364
1	303 078	151 539	597 354	298 677
2	57 105	19 035	52 923	17 641
3	20 540	5 135	5 996	1 499
4	5 040	1 008	1 255	251
5	1 374	229	348	58
6	189	27	189	27
7	56	7	48	6
8	36	4	18	2
9	10	1	20	2
10	0	0	11	1
