# Supplementary material for: Equivalent Indels – Ambiguous Functional Classes and Redundancy in Databases
Source: PLoS One. 2013 May 2;8(5):e62803. doi: 10.1371/journal.pone.0062803 (PMC3642179; doi:10.1371/journal.pone.0062803)
Supplement: Table S2 — The table presents the number of indels of a specific length. In the column #all ambiguous the number of all ambiguous indels of this length are written. The column #ambiguity classes indicates, how many indels have the specific length if two equivalent variations are put together in one class. (PDF) [file pone.0062803.s002.pdf]

Table S2. The table presents the number of indels of a specific length. In the column *#all ambiguous* the number of all ambiguous indels of this length are written. The column *#ambiguity classes* indicates, how many indels have the specific length if two equivalent variations are put together in one class.

	**Deletions**		**Insertions**	
length [bp]	#all ambiguous	#ambiguity classes	#all ambiguous	#ambiguity classes
1	33 682	15 521	331 313	161 809
2	72 718	36 081	120 913	57 102
3	34 127	16 269	49 095	23 479
4	103 408	46 059	83 279	40 047
5	19 066	8 882	23 185	11 207
6	36 090	14 266	14 598	7 043
7	4 386	2 056	4 526	2 192
8	32 010	12 801	9 725	4 679
9	5 214	2 257	3 403	1 659
10	10 722	5 114	3 845	1 852
11	2 065	1 017	1 455	723
12	6 010	2 933	2 729	1 346
13	1 436	711	1 103	550
14	3 300	1 631	1 072	533
15	1 669	825	905	451
16	3 538	1 756	1 068	533
17	829	411	690	343
18	2 361	1 171	632	315
19	678	338	522	260
20	2 201	1 087	546	272
21	592	294	352	175
22	1 221	607	295	147
23	480	236	233	116
24	1 356	668	392	193
25	388	192	200	100
26	884	436	191	95
27	341	170	163	81
28	715	353	186	93
29	231	114	106	53
30	592	292	122	61
31	177	88	109	54
32	427	211	94	47
33	108	53	74	37
34	287	142	88	44
35	93	46	51	25
36	143	71	136	68
37	40	20	107	53
38	100	50	101	50
39	28	14	74	37
40	65	32	121	59
41	34	17	64	32
42	48	24	79	39
43	26	13	32	16
44	36	18	68	34
45	24	12	44	22
46	29	13	10	5
47	22	10	14	7
48	55	24	16	8
49	8	4	0	0
50	26	11	6	3

	Deletions		Insertions	
length [bp]	#all ambiguous	#ambiguity classes	#all ambiguous	#ambiguity classes
51	14	6	8	4
52	48	19	2	1
53	21	10	4	2
54	55	24	0	0
55	47	20	0	0
56	45	20	0	0
57	27	12	0	0
58	27	12	2	1
59	21	10	0	0
60	44	17	0	0
61	41	18	0	0
62	16	7	0	0
63	29	13	0	0
64	52	22	0	0
65	33	15	2	1
66	39	17	2	1
67	24	11	0	0
68	32	14	0	0
69	27	12	0	0
70	33	14	2	1
71	15	7	0	0
72	25	12	0	0
73	15	7	2	1
74	26	11	0	0
75	21	10	2	1
76	24	10	0	0
77	23	9	0	0
78	22	10	2	1
79	30	13	2	1
80	12	6	0	0
81	7	3	0	0
82	24	11	0	0
83	5	2	0	0
84	27	13	0	0
85	11	5	0	0
86	19	7	0	0
87	11	4	0	0
88	11	5	0	0
89	10	5	0	0
90	33	15	0	0
91	11	5	0	0
92	17	7	0	0
93	21	10	0	0
94	8	4	0	0
95	20	8	0	0
96	20	9	0	0
97	9	4	0	0
98	9	4	0	0
99	5	2	0	0
100	16	6	0	0
