# Supplementary figures and images for: Equivalent Indels – Ambiguous Functional Classes and Redundancy in Databases
Source: PLoS One. 2013 May 2;8(5):e62803. doi: 10.1371/journal.pone.0062803 (PMC3642179; doi:10.1371/journal.pone.0062803)

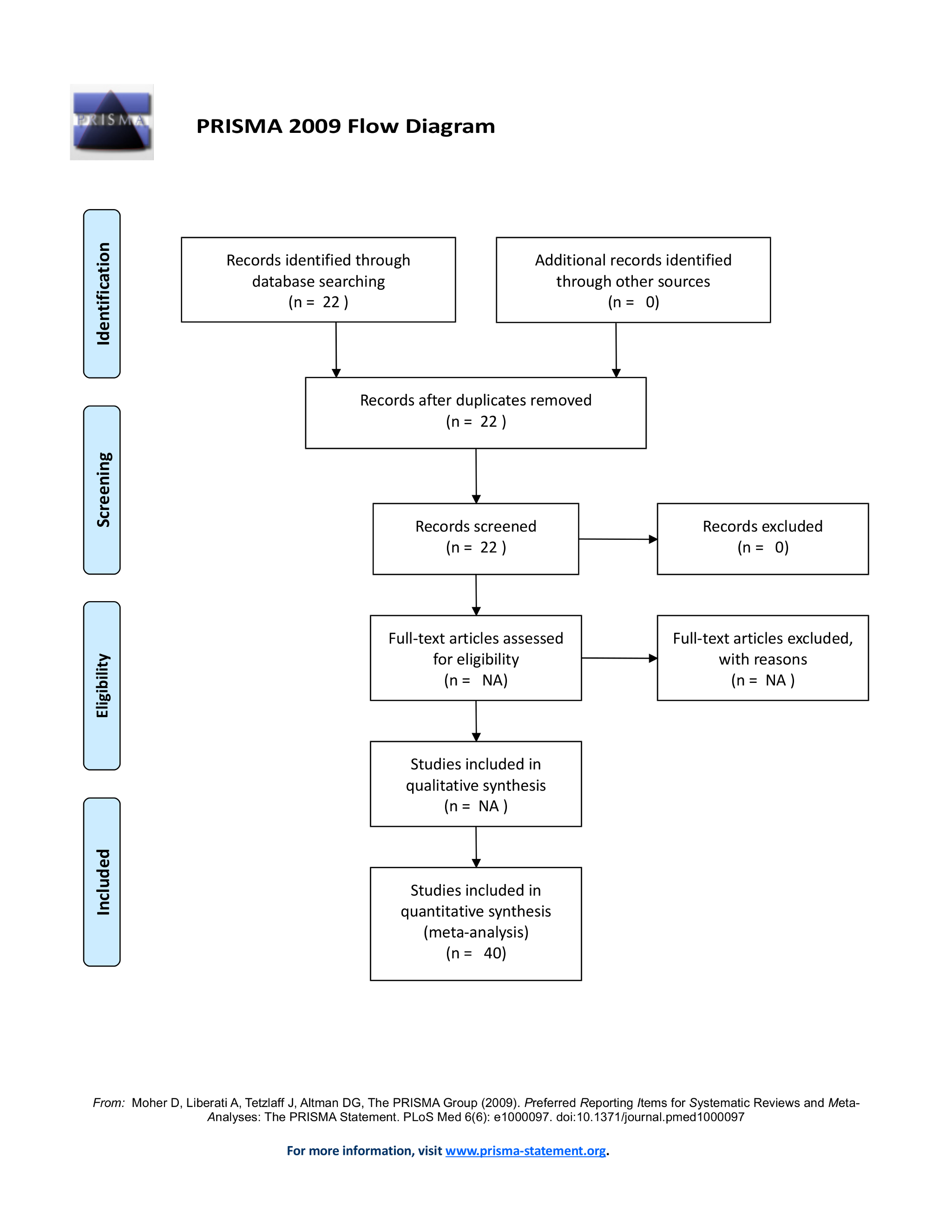

Supplement: Flowchart S1 — PRISMA Flowchart. (TIF) [file pone.0062803.s005.tif]
